# Effects of ice and floods on vegetation in streams in cold regions: implications for climate change

**DOI:** 10.1002/ece3.1283

**Published:** 2014-10-12

**Authors:** Lovisa Lind, Christer Nilsson, Christine Weber

**Affiliations:** 1 Landscape Ecology Group, Department of Ecology and Environmental Science, Umeå UniversitySE-901 87, Umeå, Sweden; 2 Eawag: Swiss Federal Institute of Aquatic Science and TechnologySeestrasse 79, CH-6047, Kastanienbaum, Switzerland

**Keywords:** Anchor ice, climate change, in-stream mosses, northern Sweden, plants, riparian vegetation, streams, winter floods

## Abstract

Riparian zones support some of the most dynamic and species-rich plant communities in cold regions. A common conception among plant ecologists is that flooding during the season when plants are dormant generally has little effect on the survival and production of riparian vegetation. We show that winter floods may also be of fundamental importance for the composition of riverine vegetation. We investigated the effects of ice formation on riparian and in-stream vegetation in northern Sweden using a combination of experiments and observations in 25 reaches, spanning a gradient from ice-free to ice-rich reaches. The ice-rich reaches were characterized by high production of frazil and anchor ice. In a couple of experiments, we exposed riparian vegetation to experimentally induced winter flooding, which reduced the dominant dwarf-shrub cover and led to colonization of a species-rich forb-dominated vegetation. In another experiment, natural winter floods caused by anchor-ice formation removed plant mimics both in the in-stream and in the riparian zone, further supporting the result that anchor ice maintains dynamic plant communities. With a warmer winter climate, ice-induced winter floods may first increase in frequency because of more frequent shifts between freezing and thawing during winter, but further warming and shortening of the winter might make them less common than today. If ice-induced winter floods become reduced in number because of a warming climate, an important disturbance agent for riparian and in-stream vegetation will be removed, leading to reduced species richness in streams and rivers in cold regions. Given that such regions are expected to have more plant species in the future because of immigration from the south, the distribution of species richness among habitats can be expected to show novel patterns.

## Introduction

Riparian zones are dynamic, diverse, and fundamentally important landscape components in most parts of the world (Gregory et al. 1991; Naiman and Décamps 1997; Morris et al. 2002). Their dynamics are usually seen as a result of the flow regime during the ice-free period, in cold regions characterized by spring floods, summer low flows, and floods triggered by rainstorms in the autumn. Flooding affects plant growth directly by reducing respiration and photosynthesis during inundation (Van Eck et al. 2006), and indirectly by determining soil texture through erosion and sedimentation (Henry et al. 1996). Floods can also cause physical injury to plants through scarring, bending, and uprooting (Kozlowski 1997). Species tolerance to flooding is therefore reflected in the zonation along elevation gradients in relation to the stream channel, with more flood-tolerant species at low elevations (Auble et al. 1994; Nilsson 1999; Van Eck et al. 2004). Vegetation at low elevations often consists of annuals and biennials, and at higher levels of perennial, woody plants (Uunila 1997; Prowse and Culp 2003). However, as an interface between aquatic and terrestrial environments, riparian zones are also exposed to ice action (Lind et al. 2014).

Streams in cold regions are subjected to specific hydrological processes that control flow regime, water levels, and ultimately biota (Hicks 2009; Stickler et al. 2010). The most important processes include the formation, growth, and breakdown of river ice during the winter, which represents a significant part of the year (Prowse and Beltaos 2002; Luke et al. 2007). By creating obstacles to flow, stream and river ice can produce winter floods with magnitudes often exceeding those created by open-water conditions (Prowse and Beltaos 2002). The channels are characterized by different types of ice, and its formation depends upon variation in conditions such as local flow, meteorology, and topography (Stickler and Alfredsen 2005). An ice cover can be formed in either a static or dynamic manner. A stable surface ice cover is most common in reaches with low velocity, whereas a dynamic ice cover characterizes more turbulent reaches (Beltaos 2013). In stream and river systems in cold regions, dynamic ice production is common when the air is cold and the water super-cooled (Stickler and Alfredsen 2005). Frazil ice (tiny ice crystals; Martin 1981) forms near the water surface, during super-cooled conditions, and can be transported to the streambed where it attaches to submerged objects such as boulders and vegetation, building up anchor ice and occasionally anchor-ice dams (Stickler and Alfredsen 2009). As the temperature rises, anchor ice detaches, drifts downstream and often accumulates and may again form anchor-ice dams (Stickler and Alfredsen 2009). Anchor-ice dams, while building up, can cause flooding of riparian zones as the water level rises and the flow velocity decreases (Stickler et al. 2010). When the anchor-ice dam eventually breaks due to elevated water stage and a higher pressure, it increases the flow velocity and the potential for physical disturbance to the riparian zone. As for floods during ice-free seasons, ice and ice-induced floods exert stress and disturbance to riparian and in-stream vegetation (Prowse 2001; Rood et al. 2007; Lind et al. 2014). However, a main difference is that ice-related floods not only exert a physiological stress but also expose the vegetation to physical disturbance from freezing in ice and scouring from moving ice (Lind et al. 2014).

It has been recognized that, as a result of ongoing climate change, northern regions will be exposed to a greater temperature increase than the global average (Andréasson et al. 2004). In such regions, changes in temperature and precipitation during winter will result in more frequent shifts between ice thawing and freezing (Mote et al. 2003; Andréasson et al. 2004). Winter is perceived as a bottleneck in the life history of plants and animals and the effects of a changing climate can be manifold (Beltaos 2013). As part of continued climate change, a stable ice cover may not even develop in many streams in cold regions (Prowse and Beltaos 2002), exposing the in-stream biota to open-water conditions and reducing the disturbance of riparian vegetation. Climate change may also influence the Atlantic meridional overturning circulation, which could lead to an increase in ice production as the temperature decreases (Bryden et al. 2005). As the riparian zone provides many ecosystems services, such as nutrient retention and biodiversity (Nilsson and Renöfält 2008), it is important to investigate its response to a changing climate. Today, there is growing interest in research on the dynamics of stream and river ice as the effects of climate change on ice may have substantial economic and ecological consequences (Beltaos and Burrell 2003).

To increase the ability to predict species responses to future climate change, it is important to compare the plant composition among streams and rivers that differ in ice regime. We studied how different types of vegetation are impacted by different types of ice and winter floods. Specifically, we investigated how anchor ice and consequently winter floods influence the species composition of the riparian vegetation, and how the presence of ice affects the survival rate of vascular plants and in-stream mosses. Our study consisted of three parts. In the first part, we applied future climate scenarios to the study streams and identified four sets of geographically close, boreal streams that would represent two different stages in the predicted development of future ice regimes. It is, however, difficult to foresee changes in ice dynamics, for example where and when floods and anchor-ice dams will develop during winter (Rood et al. 2007). Therefore, in the second part, we exerted physiological stress and physical disturbance to the vegetation by watering riparian vegetation during cold weather. We mimicked two types of mid-winter flood events. In the first experiment, we exposed plots with riparian vegetation to flooding several times during winter to evaluate the effects of multiple flood events. In the second experiment, plots with riparian vegetation were exposed to a single extensive flood event. In the third part of the study, we quantified the physical force of naturally moving ice on riparian and in-stream vegetation using plant and moss mimics as meters. Specifically, we stuck wooden sticks in the riparian ground and attached moss transplants to in-stream boulders, and measured to what degree the sticks and transplants were eroded in streams with different ice regimes.

## Materials and Methods

### Study area

**Figure 1:**
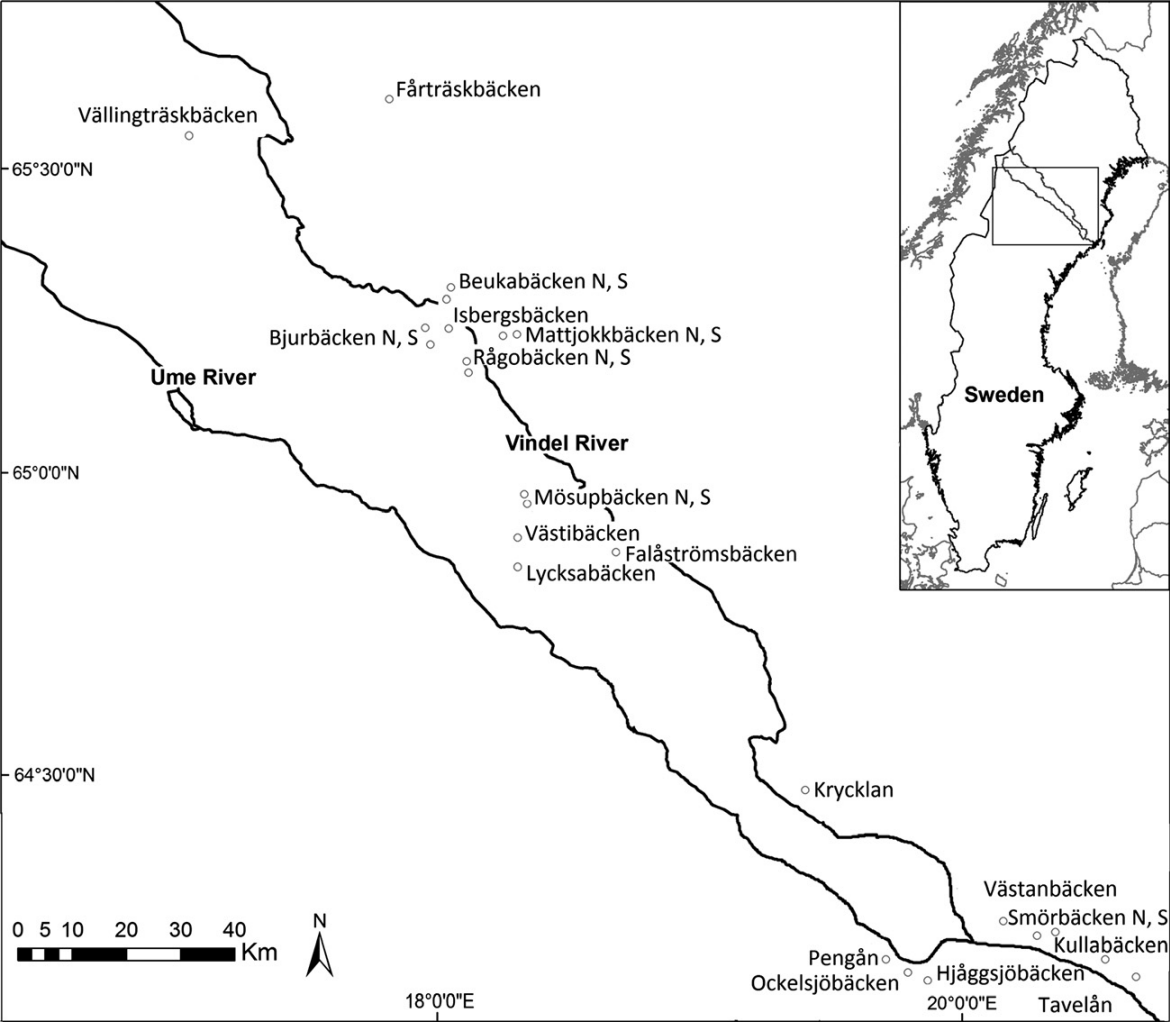
Detailed map showing the location of study reaches along the Vindel and Ume rivers in Västerbotten county, northern Sweden. Twenty-five study reaches were distributed along tributaries to the rivers between the Scandes Mountains and the coast. Inserted map shows the whole of Sweden with the location of the Ume and Vindel rivers. S = south, N = north.

The study reaches were located in the county of Västerbotten, northern Sweden. The reaches were situated in tributaries to the Ume and Vindel rivers, which originate in the Scandes Mountains and empty into the Gulf of Bothnia (Fig. 1). The Vindel and Ume rivers flow through a landscape formed by glaciations and crustal rebound, with till deposits dominating above and lacustrine sediment below the former highest coastline, which is formed by a pre-stage of the Baltic Sea (about 10,000 years ago). A total of 25 reaches were included in the winter survey, and reaches for the different experiments were selected among these. The study streams have their spring flood peaks due to snowmelt in April–June. The riparian vegetation in a transverse profile across the study reaches is zoned with forest communities at the highest topographical position, followed by shrubs, and various graminoid and forb communities at the lower position (Nilsson 1999). *Carex* spp. are the most abundant graminoids, whereas *Vaccinium* spp. dominate the highest riparian elevations and the uplands. The boreal forest is dominated by *Picea abies*, *Pinus sylvestris*, *Betula pubescens*, and *Alnus incana*.

### Winter survey

**Figure 2:**
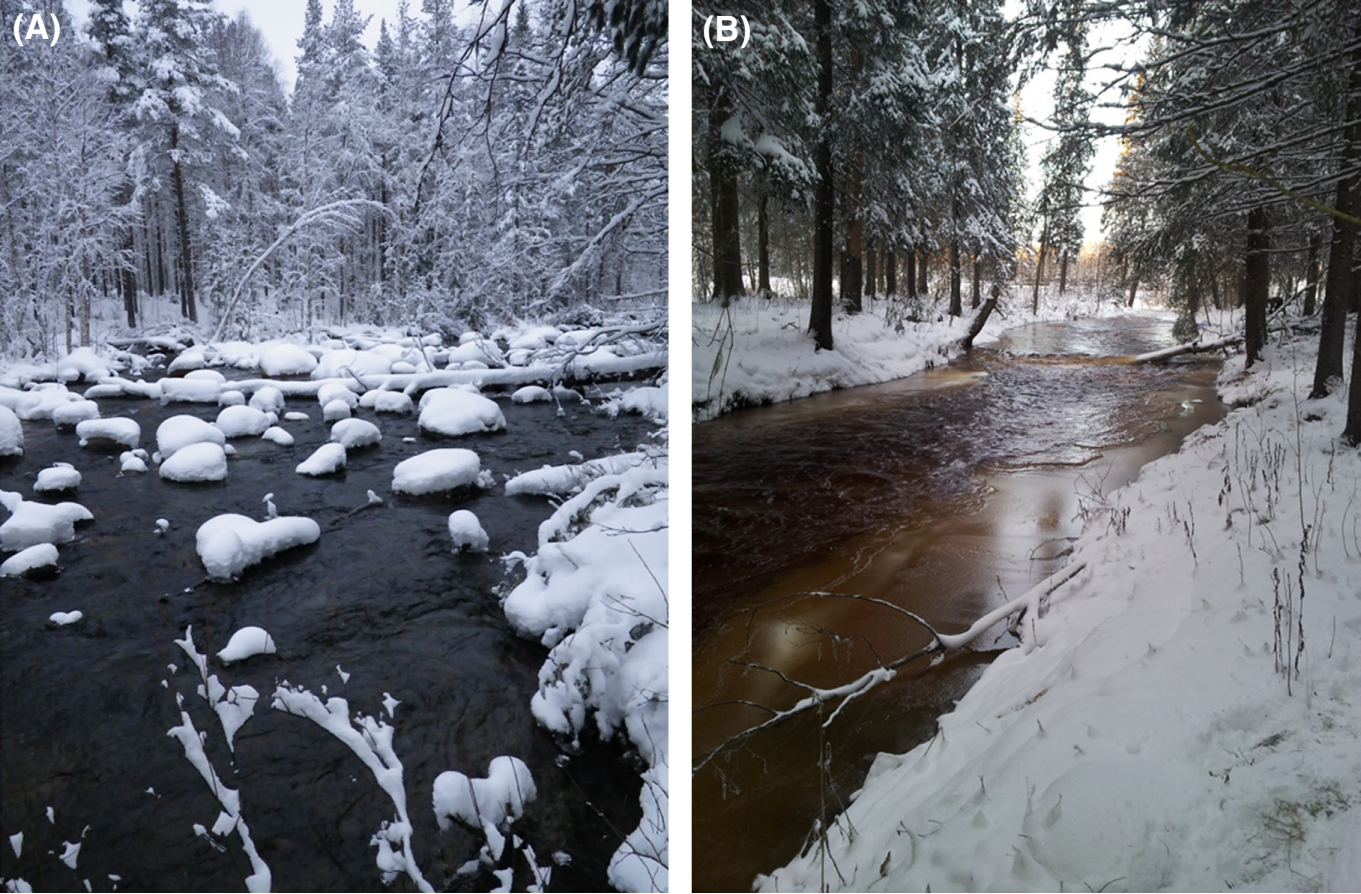
Examples of study reaches that are anchor-ice free (A) and anchor-ice rich (B).

Along a 200-m section of each of the 25 reaches, the spatial extent of anchor ice, surface ice, and aufeis (formed when water is forced through the surface ice layer and progressively freezes onto the original layer) was mapped and photographed six times between November and April during each of two consecutive years (2011–2013). Specific ice formations (e.g., anchor-ice dams and aufeis) were also indicated on the maps. To identify temporal changes in early and late winter, ice formation was surveyed using Wingscapes Time Lapse Plant Cameras, which were permanently placed at each reach. Cameras were set to take three pictures per day from October to the end of April. The cameras were, however, not reliable at temperatures below −15°C. Based on the winter survey, the reaches were classified into three different groups based on the maximum spatial extent of anchor-ice cover of the entire wetted areas; one anchor-ice-rich group, with a high spatial extent of anchor-ice formation in the study reach (>30% anchor-ice cover), one with low spatial extent of anchor ice (5–30%), and another that lacked an anchor-ice cover (0–5%; Fig. 2; Table 1).

**Table 1:** Description of study reaches and in which studies they were included.

Nr	Stream	Maximum anchor-ice cover (%)	Altitude (MSL)	Part 1	Part 2		Part 3	
		
				Future climate scenarios	Multiple floods	Extended flooding	Mimicking vascular plants	Mimicking in-stream mosses
1	Smörbäcken S	>30	120		X		X	
2	Hjåggsjöbäcken	0–5	140		X		X	
3	Ockelsjöbäcken	0–5	179		X		X	
4	Kullabäcken	0–5	70		X	X	X	
5	Västanbäcken	>30	120		X	X	X	
6	Tavelån	>30	39		X	X	X	
7	Smörbäcken N	0–5	128	X	X	X	X	
8	Pengån	>30	95	X	X		X	
9	Mattjokkbäcken N	>30	271	X			X	X
10	Beukabäcken S	0–5	298	X			X	X
11	Lycksabäcken	>30	239	X			X	
12	Fårträskbäcken	>30	385	X			X	
13	Rågobäcken N	0–5	293	X			X	
14	Västibäcken	0–5	296	X			X	
15	Falåström	0–5	247				X	
16	Beukabäcken N	0–5	300				X	
17	Rågobäcken S	>30	264				X	
18	Isbergsbäcken	0–5	304				X	
19	Krycklan	>30	193				X	
20	Mattjokkbäcken S	>30	276				X	
21	Bjurbäcken S	0–5	314				X	
22	Bjurbäcken N	0–5	321				X	
23	Mösupbäcken S	>30	291				X	
24	Mösupbäcken N	5–30	290				X	
25	Vällingträskbäcken	>30	304				X	

### Future climate scenarios

Data from climate models for 1961–2099 for the Ume/Vindel River drainage area were obtained from the Swedish Meteorological and Hydrological Institute (SMHI). The obtained data included winter temperature (°C), winter precipitation (mm), number of days during winter with temperatures both below and above zero, that is, *T*_max_ > 0°C and *T*_min_ < 0°C, and length of growing season, that is, number of days with an average temperature >5°C. The meteorological period of 1961–1991 was used as a validation and a reference for the models. The mean values of change in obtained data were based on calculations from nine global climate models under the RCP 8.5 and RCP 4.5 scenarios (CCCma-CanESM2, CNRM-CERFACS-CNRM-CM5, ICHEC-EC-EARTH, IPSL-IPSL-CM5A-MR, MIROC-MIROC5, MOHC-HadGEM2, MPI-M-MPI-ESM-LR, NCC-NorESM1-M, NOAA-GFDL-GFDL-ESM2M; SMHI 2014). The RCP 8.5 scenario is based on an increase in greenhouse gas emissions, whereas the RCP 4.5 scenario assumes reduced greenhouse gas emissions.

Vegetation was inventoried in summer along four reaches that represent a future scenario of anchor-ice-free (0–5%) and four that represent a future scenario of anchor-ice-rich reaches (>30%; Table 1: numbers 7–14). Five transects were spaced at 10 m intervals along each reach, with plots (50 × 50 cm) at three elevations, (0, 40, and 80 cm) above the stream channel at summer low flow, to cover the whole riparian zone. The inventory included the percent cover of all vascular and nonvascular plants (<2 m high) rooted inside the plots, and the percent cover of bare soil and boulders, woody debris, and standing water, respectively. In some cases, two or more species were treated as one taxon: *Carex juncella + C.nigra*, *Galium* spp., *Hieracium* spp., *Salix myrsinifolia* + *S. phylicifolia*, *Sparganium* spp., and *Taraxacum* spp. Bryophytes included mosses and liverworts. The vegetation cover was summarized as cover of grasses, forbs, and woody plants, respectively. To evaluate differences in cover of these functional groups in relation to presence of ice, altitude in meter above sea level (MSL), elevation above the stream channel, and substrate (bare soil, boulders, woody debris and standing water), we used generalized linear models (GLM) with quasi-Poisson distribution to correct for overdispersion. All analyses were performed using R version 2.15.2 (R Development Core Team 2012), if not stated otherwise.

### Multiple floods

**Figure 3:**
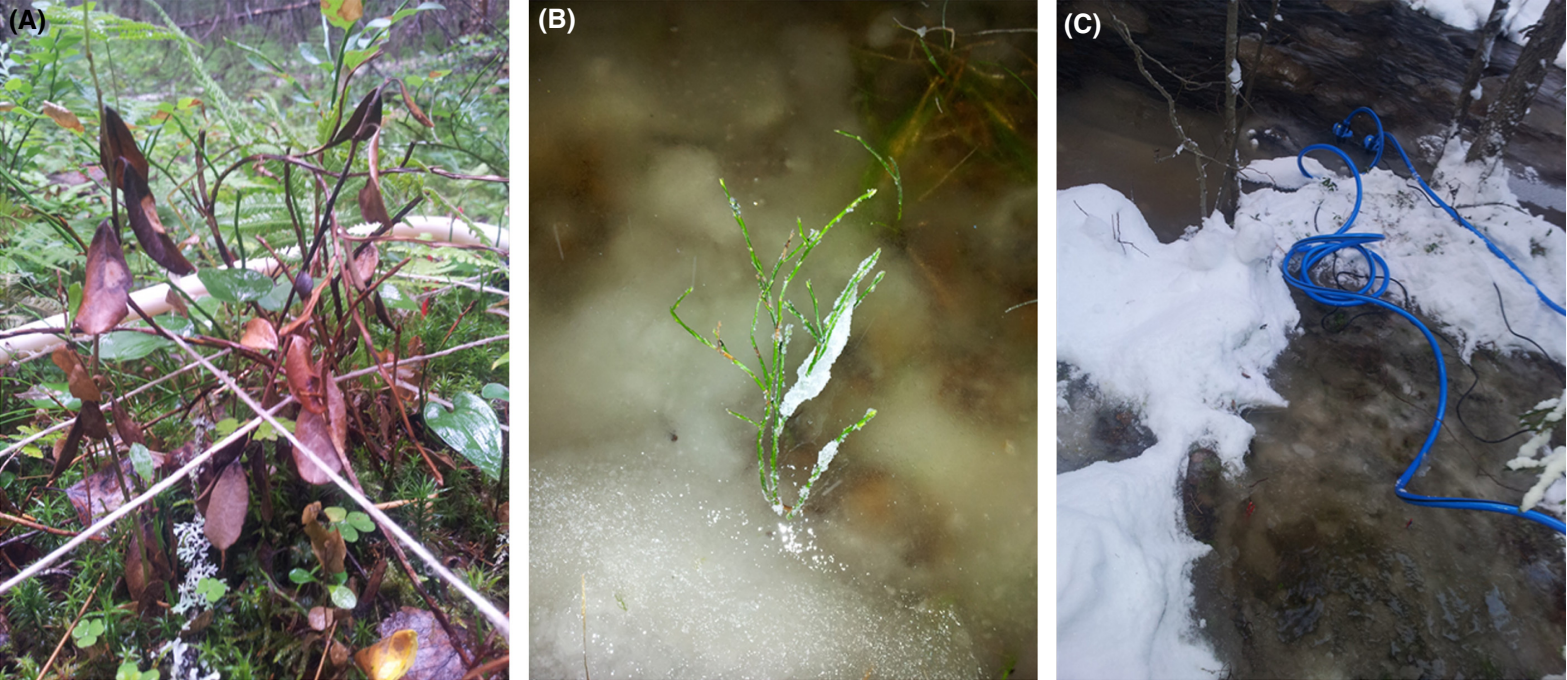
Flooding experiment in the riparian zone. (A) *Vaccinium vitis-idaea* in the summer following the extended flooding experiment, (B) *Vaccinium myrtillus* protruding above the experimentally created ice cover, and (C) Water pumping over the riparian zone.

To study the effects of multiple floods during winter, we used our set of study reaches to select four anchor-ice-rich reaches (>30%) and four anchor-ice-free reaches (0–5%; Table 1: numbers 1–8). The reaches were located in tributaries to the Ume River and were situated within 40 km from the city of Umeå (Fig. 1). In each reach, four turbulent sections were selected as the production of frazil and anchor ice is higher in turbulent than in tranquil sections. Two different riparian elevations (40 and 80 cm above the summer low flow) were identified in each section, using a clinometer and a rod. To create an ice cover through watering by hand, 15-cm-high circular plastic frames were anchored to the ground to keep water from flowing out. To be able to anchor the frames, the plots were restricted to a diameter of 25 cm. The plots were placed in the riparian zone in the middle of each turbulent section, and eight plots were located at each elevation. To find the plots under snow, this was gently removed before watering, and replaced afterward. In each plot, 30 L of water was slowly poured inside the frames until the whole water volume was absorbed and an ice layer started to form. The watering was conducted during 4 days distributed between November and February in the winter 2011–2012, during which time temperatures remained below −10°C. The vegetation cover in the plots was quantified before the experiment, in July–August 2011. Four control plots at each elevation and section, not subjected to the experimental watering, were also inventoried. Vegetation was inventoried by percent cover of species according to the same premises as previously described in the methods section for future climate scenarios. In the summer 2012, the plots were re-inventoried, and visible vegetation damage such as frost burns was recorded (Fig. 3A and B).

To evaluate changes in species composition before and after the multiple flooding experiments, we used a nonparametric multi-response permutation procedure (MRPP), to test effects of treatment between groups. The test was run for three different sets of groups. We defined the sets of groups as (1) plots along anchor-ice-rich and anchor-ice-free reaches before versus after the watering treatment, to separate the treatment effect from the natural disturbance; (2) plots from anchor-ice-rich versus anchor-ice-free reaches after watering treatment; and (3) untreated control plots from 2011 versus 2012 to account for in-between year differences. The MRPP provides the test statistic *T,* which is more negative the stronger the separation is between the groups. It also provides a *P*-value, associated with *T*. The MRPP also supplies a description of the effect size, independent of the sample, by the chance-corrected within-group agreement (*A*). *A* describes within-group homogeneity compared to the random exceptions: *A* < 0 with more heterogeneity within groups than expected by chance; *A* = 0 when heterogeneity within groups equals expectation by chance; *A* = 1 when all items are identical within groups.

Analysis of species richness and multivariate analyses were performed in PC-Ord version 6.0. Differences in species richness were analyzed using analysis of variance (ANOVA). Analyses were also applied on the control plot data to exclude interannual differences. To evaluate the effects on riparian bryophytes exposed to the flooding experiment, we used linear mixed-effects models (LME) with two random factors that nested the plots within elevation and reach. The same type of analysis was used to compare the cover of different functional groups before and after the flooding experiments and in the control plots, with stream as a random factor. The R package nlme was used in the mixed-effects models analysis (Pinheiro et al. 2013). All analyses were performed using R version 2.15.2 (R Development Core Team 2012), if not stated otherwise.

### Single extended flood

To study the combined effect of flowing water and freezing, we created extended flooding by pumping water from the stream to the riparian area during cold (<−15°C), snow-free days in early winter. The experiment was made in four of the reaches that were included in the multiple flooding experiment (Table 1: numbers 4–7). Plots were placed in the riparian zone in the middle of each turbulent section. Watering was applied at each reach in four plots (50 × 50 cm) at two elevations (40 and 80 cm above the summer low water level). The size of the plots was larger than in the multiple floods experiment as the number of plots was restricted by the use of water pumps, whereas the size was not restricted by any use of frames. During 4 h, which was constrained by cold weather and the short days, about 3200 L/h of water was pumped from the stream (Fig. 3C). The plots were inventoried before the experiment, in July–August 2012 and re-inventoried in the summer 2013. Four control plots at each elevation were also inventoried. All vegetation inventories and statistical analysis were preformed according to the same premises as described in the methods for the multiple floods experiment.

### Mimicking in-stream mosses

To evaluate the effects of the physical disturbance of flow and ice on bryophytes, we used transplants of the dominant in-stream moss in boreal areas, *Fontinalis* spp., collected in reaches not included in any other study. The samples were washed to remove any epiphytes or detritus, dried, weighed, and then affixed to Velcro strips (5 × 7 cm). The moss mimics were thereafter attached to boulders in Mattjokkbäcken N and Beukabäcken S (Table 1: numbers 9–10). The reach in Mattjokkbäcken is anchor-ice rich (>30% cover), whereas the reach in Beukabäcken is anchor-ice free (0–5% cover). The two streams were restored in 2010 whereby boulders from adjacent upland heaps were replaced to the channel; the boulders were therefore missing a natural, in-stream bryophyte community. Strong instant glue was used to secure the Velcro strips onto the upstream and downstream sides of 15 boulders in each stream in turbulent reaches at low flow. Bryophytes have a large capacity of regaining strength after drying when being re-wetted (Csintalan et al. 1999); however, some loss of strength was expected. Current velocity was measured during summer low flow both upstream and downstream of the boulders using a hand-held velocity recorder (Valeport, Model 801). After one winter, the Velcro strips with the remaining *Fontinalis* were collected. The *Fontinalis* transplants were then removed from the Velcro strip and again dried in 65°C to constant weight. To quantify the difference in moss weight before and after the winter season along an anchor-ice-rich and an anchor-ice-free reach, we used generalized mixed-effect model (GLM) with Poisson distribution as the data were not normally distributed. Position on boulder (upstream or downstream) and current velocity at each boulder were also included as factors in the analysis. All analyses were performed using R version 2.15.2 (R Development Core Team 2012).

### Mimicking vascular plants

In this part of the study, all 25 reaches were included (Table 1: numbers 1–25). We used bamboo sticks to mimic vascular plants during two winters, 2011–2013 (Dong et al. 2001; Kohler et al. 2004; Tsujino and Yumoto 2008). Ten sticks (30 cm long, 5 mm thick) were placed in 15 plots, which were evenly spread with a 10 m interval and among three elevations, (0, 40, and 80 cm) above the stream channel at summer low flow. Sticks were pushed 10 cm down in the substrate along all reaches. In the following year, the sticks were counted and categorized as broken, missing, or unimpacted. A total of 3750 sticks/year were used in the study, meaning that 1250 sticks were placed at each elevation. During the second study year, one of the reaches was affected by logging and therefore excluded from the study. To evaluate the effects of anchor ice on plant mimics (sticks), we used LME with two random factors that nested the sticks within elevation and stream. All analyses were performed using R version 2.15.2 (R Development Core Team 2012).

## Results

### Future scenarios

**Figure 4:**
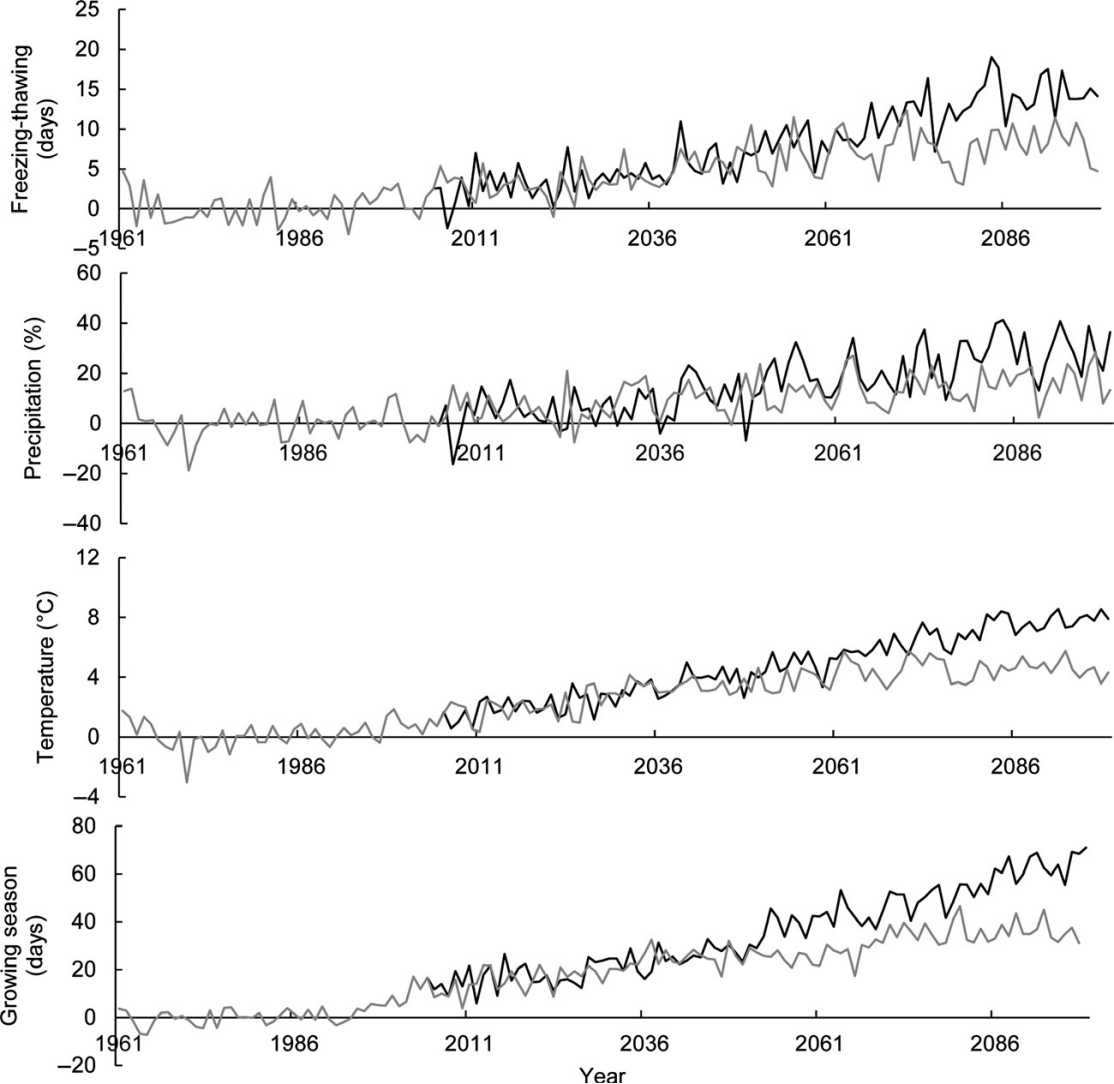
Calculated change for 1961–2099 in winter temperature (°C), precipitation (%), number of shifts between freezing and thawing (days), and length of growing season (days) for the Ume and Vindel rivers drainage area, in which mean values from 1961 to 1991 were used as validation and reference. Mean values were based on calculations from nine global climate models under RCP 8.5 scenario (black line) and RCP 4.5 scenario (gray line).

Mean values from the nine climate models for both scenarios (RCP 8.5 and RCP 4.5) show an increase in precipitation, temperature, days with shifts between freezing and thawing, and length of growing season. Scenarios are projected for the Ume/Vindel River drainage area, and the increase in climate variables has a steeper incline in the RCP 8.5 scenario than in the RCP 4.5 scenario (Fig. 4). The average winter temperature for the whole drainage area is −11.6°C (December, January, and February: 1961–1991). With scenario RCP 4.5, the changes in temperature will result in an average winter temperature of −7.3°C in the year 2099, and with the RCP 8.5 scenario, in an average winter temperature of −3.7°C in 2099.

**Figure 5:**
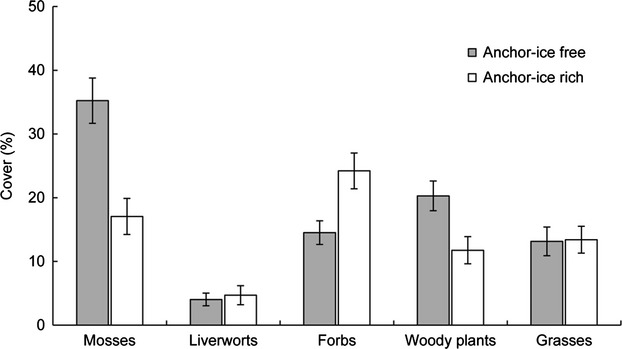
Percent cover (±1 SE) of functional groups of vegetation (mosses, liverworts, grasses, forbs, and woody plants) in the riparian zone representing a scenario of anchor-ice-rich (>30% cover) and anchor-ice-free (0–5% cover) streams. Forbs had a significantly higher (Est. = 1.2, *P* = 0.00052) cover in anchor-ice-rich streams than in anchor-ice-free streams, whereas riparian mosses showed the opposite pattern (Est. = −2, *P* = 0.0005).

The climate change scenarios cover an altitudinal gradient (mountain to coast), which represents substantial differences in ice regimes, ranging from streams with a stable ice cover to ice-free streams. As an illustration of extreme cases, we let four streams represent an anchor-ice-rich, and four streams an anchor-ice-free situation. An inventory of the streams showed that these differences also played a role in terms of plant community composition. The anchor-ice-rich streams had a higher cover of forbs in the riparian zone (Estimate = Est.) = 1.2, *P* = 0.00052), whereas a different pattern was shown for the anchor-ice-free streams (Fig. 5). Mosses were more common in anchor-ice-free streams (Est. = −2, *P* = 0.0005), whereas for grasses, woody plants, and liverworts the altitude (MSL) of the stream reach was more important for their cover than the presence of ice (*P* < 0.05). Substrate and elevation above the stream channel at summer low flow did not show any relationship with vegetation cover and were not included in the final model. Anchor-ice-rich streams had a higher cover of the forb *Filipendula ulmaria* (4.5% vs. 1.4%), whereas the cover of the woody shrub *Vaccinium myrtillus* was higher along anchor-ice-free streams (8.2% vs. 1.05%).

### Multiple floods

**Figure 6:**
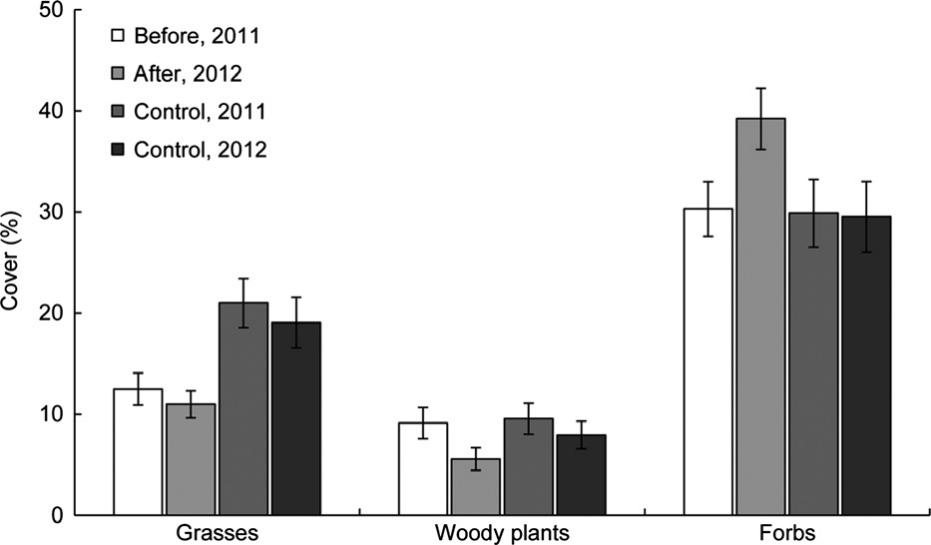
Percent cover (±1 SE) of functional groups of vegetation (grasses, woody plants, and forbs) in the riparian zone, before and after experimentally created multiple winter flooding events and in unwatered control plots for 2011 and 2012. Forbs had a significantly higher cover (*F* = 8.02, df = 230, *P* = 0.005) and woody plants had a significantly lower cover (*F* = 3.51, df = 230, *P* = 0.031) after the experiment. There were no differences in the control plots (*P* > 0.05) between the years.

The multiple floods experiment caused visible damage to stems and leaves of *V. myrtillus*, *Vaccinium vitis-idaea*, and *Luzula pilosa*, which turned black and died (Fig. 3A and B). In the following summer, species richness had increased significantly (Table 2). The community composition (percent cover) of vascular plants differed between anchor-ice-rich and anchor-ice-free reaches (*T* = −2.312, *A* = 0.048, *P* = 0.023; data from all elevations for treated plots summed), but the plant community composition was not affected by the experiment (*T* = 1.634, *A* = −0.033, *P* = 0.99). Neither were there any differences in the plant community composition in the control plots in-between years (*T* = −0.253, *A* = 0.0005, *P* = 0.331; Table 3). Bryophytes remained unaffected by the multiple floods. However, when functional groups of plants were considered, forbs had a higher cover after the experiment (*F* = 8.02, df = 230, *P* = 0.005) and woody plants had lower cover (*F* = 3.51, df = 230, *P* = 0.031), whereas grasses were unaffected by the multiple floods. In the unwatered control plots, there were no significant changes in cover in any of the functional groups between the 2 years (*P* > 0.5; Fig. 6). At the species level, there were some changes in cover in the experimental plots. *F. ulmaria* showed a 9% increase, *Equisetum* spp. (*E. arvense, E. pratense* and *E. sylvaticum*) increased by 12.3%, and *V. myrtillus* decreased by 17.3%, while there was no difference for grasses such as *Deschampsia flexuosa*. Thirteen new species had colonized the plots after the experiment, eight of which were forbs, two woody plants, and three grasses. However, four species disappeared: a forb (*Pedicularis sceptrum-carolinum*), a woody plant (*Picea abies*), and two grasses (*Milium effusum* and *Elymus caninus*).

**Table 2:** Summary of ANOVA tables showing the total species richness of plants before and after the two types of flooding experiments and their control plots.

Treatment	Before	After	*F*	df	*P*
Multiple flooding	52	61	6.3	238	0.013
Control plots	51	53	2.6	126	0.105
Extended flooding	45	53	4.7	63	0.034
Control plots	64	65	2.3	62	0.187

**Table 3:** Results of multi-response permutation procedures (MRPP) of pairwise comparisons to test for differences in riparian plant community composition among plots before and after treatment, among control plots for 2011 and 2012, and among anchor-ice-rich and anchor-ice-free reaches. A highly negative *T* represents a strong separation between the groups: *A* < 0 with more heterogeneity within groups than expected by chance; *A* = 0 when heterogeneity within groups equals expectation by chance; *A* = 1 when all items are identical within groups. Significant values are indicated in bold (*P* < 0.05).

Experiment	Treatment	*T*	*A*	*P*
Multiple flooding	Before vs. after	1.634	−0.033	0.99
	Control 2011 vs. 2012	−0.253	0.0005	0.331
	Anchor-ice free vs. rich	−2.312	0.048	**0.023**
Extended flooding	Before vs. after	2.104	0.0077	1.0
	Control 2011 vs. 2012	1.183	0.005	0.935
	Anchor-ice free vs. rich	−14.941	0.054	**<0.0001**

### Single extended flood

*Vaccinium vitis-idaea* and *V. myrtillus* showed visible damage after the flooding experiment. Leaves and stems of *Vaccinium* had turned black and died (Fig. 3A). In some plots, all plants of *V. vitis-idaea* and *V. myrtillus* showed visible frost damage, but other species remained unaffected. Species richness of vascular plants increased significantly after the extended flooding experiment, but did not differ with elevation above the stream channel. Neither was there a change in species richness in the control plots (Table 2). Overall community composition (i.e., all elevations included, for treated plots) of vascular plants also remained unchanged (*T* = 2.104, *A* = −0.0077, *P* = 1.00), neither was there a difference in the plant community composition in the control plots between the 2 years (*T* = 1.183, *A* = −0.005, *P* = 0.935), but plant community composition differed between anchor-ice-rich and anchor-ice-free reaches (*T* = −14.941, *A* = 0.054, *P* < 0.0001; Table 3). The extended flooding experiment did not affect mosses or liverworts, but there was a higher moss cover along anchor-ice-free streams (*P* = 0.0002). A look at some of the dominant species showed a 7.5% decrease of *V. myrtillus,* a 26.3% increase of *F. ulmaria*, and an 11.7% increase of *Equisetum* spp. (*E. arvense, E. pratense* and *E. sylvaticum*), but there was no difference for grasses such as *D. flexuosa*. There were no differences in cover of different functional groups (grasses, forbs, and woody plants) before and after the experiment (*P* > 0.05). However, eight new species had colonized the watered plots after the experiment, six of which were forbs and two woody plants.

### Mimicking in-stream mosses

**Figure 7:**
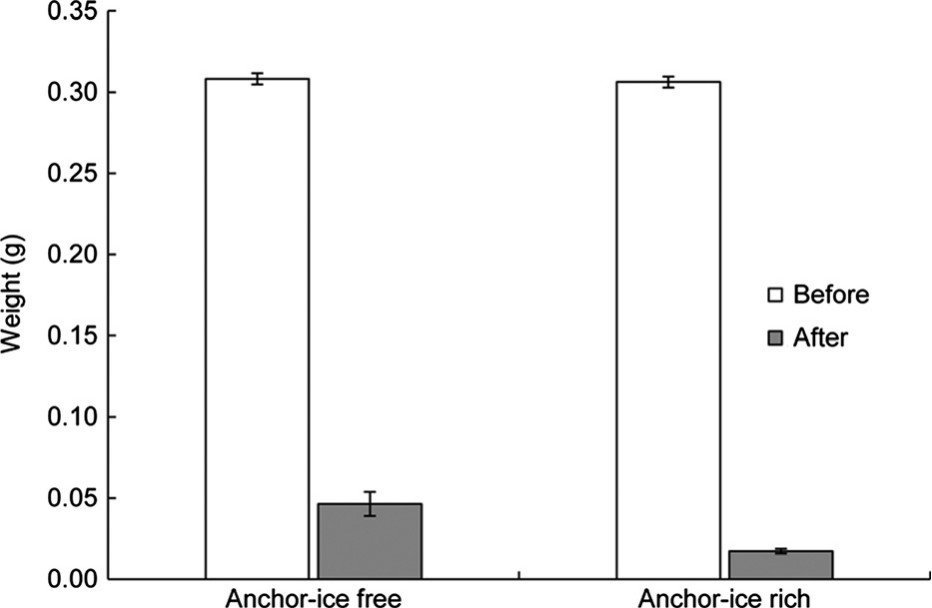
Average weight (±1 SE) of moss transplants on in-stream boulders before and after the winter 2012–2013 along an anchor-ice-rich and an anchor-ice-free stream reach. The difference before and after the winter as well as the difference between anchor-ice-rich and anchor-ice-free reaches after the winter were statistically significant (Est. = 0.88, *P* = 0.047; Est. = 0.1, *P* = 0.002).

There was a significant loss of moss transplants in both Mattjokkbäcken (anchor-ice rich) and Beukabäcken (anchor-ice free; Est. = 0.88, df = 59, *P* = 0.047). The reduction of moss transplants was larger in Mattjokkbäcken (Est. = −0.1, df = 56, *P* = 0.002; Fig. 7). The position on the boulders (upstream vs. downstream) and the current velocity did not affect the amount of moss transplants that remained after a winter (Est. = −0.0006, df = 54, *P* = 0.761 and Est. = −0.001, df = 54, *P* = 0.774).

### Mimicking vascular plants

**Figure 8:**
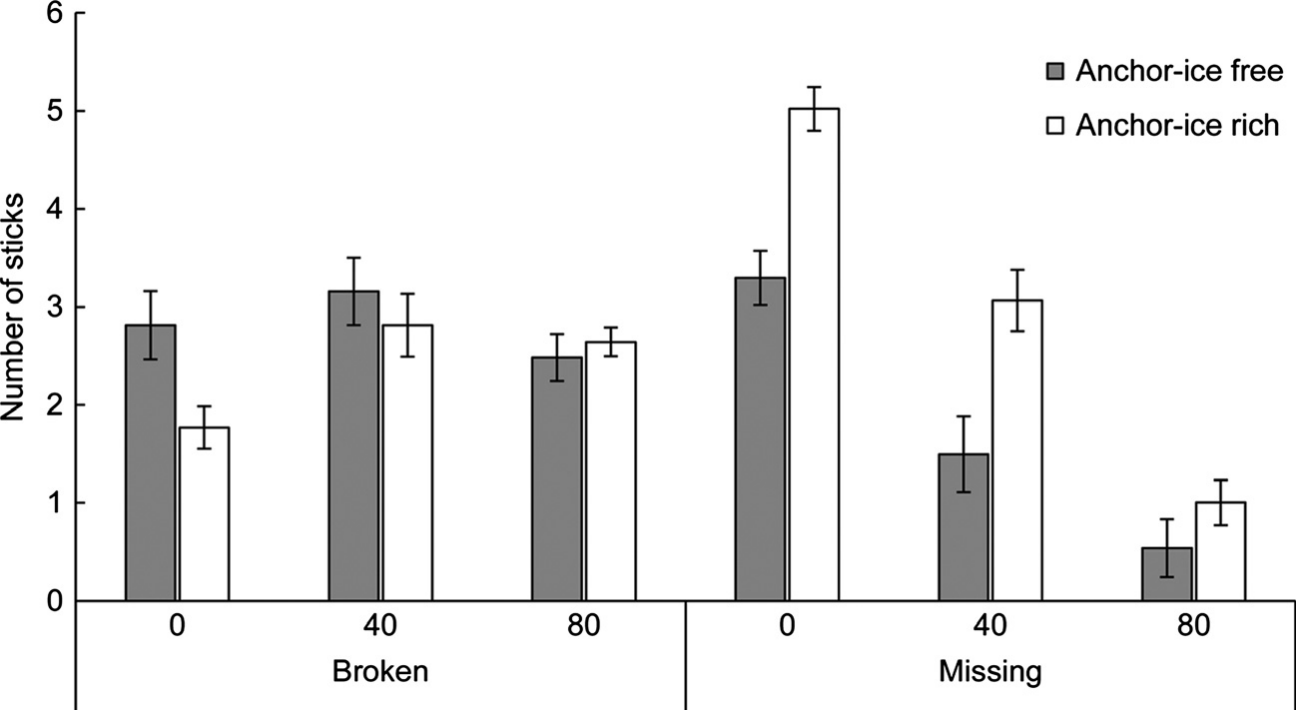
Average number of sticks (±1 SE) per plot in the riparian zone that were missing or broken after the winters 2011–2012 and 2012–2013 at three elevations (0, 40, and 80 cm) from the low water level. Anchor-ice-free reaches are shown in gray and anchor-ice-rich reaches in white. The difference in missing sticks between anchor-ice-free and anchor-ice-rich reaches was statistically significant (Est. = 0.88, df = 785, *P* = 0.047).

There were a higher number of sticks missing along ice-rich streams (Est. = 0.88, df = 785, *P* = 0.047). The number of missing sticks was also higher in plots at lower elevation, closer to the stream channel (Est. = −1.1, df = 758, *P* < 0.001; Fig. 8). A higher number of sticks were missing in 2012 than in 2013 (Est. = −0.04, df = 49, *P* < 0.001). There was no difference in the amount of broken sticks between anchor-ice-rich and anchor-ice-free reaches (Est. = −0.57, df = 786, *P* = 0.1119). Neither was there any difference in the amount of broken sticks in relation to the elevation above the stream channel (Est. = 0.006, df = 49, *P* = 0.1997).

## Discussion

Available climate change scenarios for the Ume/Vindel River catchment in northern Sweden project unanimously that especially the temperature, the number of shifts between freezing and thawing, and the length of the growing season will increase steadily over the next 80 years (Fig. 4). With an increasing number of shifts between freezing and thawing, we can foresee an initial increase in the production of frazil and anchor ice. However, as the overall air temperature is also increasing, reducing the length of winters, the production of frazil and anchor ice might decrease toward the end of this century (Beltaos et al. 2006; Nilsson et al. 2013). The formation of surface ice will be delayed as water and air temperatures increase during autumn, and the interval between the first ice formation event and the final freeze-up will lengthen (Prowse and Beltaos 2002). The entire freeze-up cycle can also change as increased flow in winter, as a result of less precipitation falling as snow, may alter the strength and the thickness of the ice cover. This would mean that more streams might stay ice-free longer, and more streams could remain ice-free during the whole year (Prowse et al. 2011).

Winter flooding and river ice breakup are important events for the water budget and the plant species richness. If the magnitude of ice breakups and the frequency of winter floods decrease, the plant composition in the riparian zone will change (Lind and Nilsson unpublished data). Available evidence suggests that an increase in anchor-ice production will lead to more species in the riparian zone, whereas a decrease and eventual disappearance of ice will reduce species richness (Engström et al. 2011). Furthermore, the proportion of forbs will be highest along anchor-ice-rich streams, whereas the proportion of dwarf-shrubs will be highest along anchor-ice-free streams. The observed differences in the proportions of forbs and dwarf-shrubs between the anchor-ice-rich and the anchor-ice-free streams support this prediction, even if the mechanisms are not revealed. We therefore made experiments to fill this knowledge gap.

It has previously been shown that plant species vary in their response to summer floods and especially longer periods of inundation (Kozlowski 1997; Van Eck et al. 2006). Our watering experiments in the riparian zone demonstrate that winter floods, most of which are induced by anchor-ice formation, can represent an important disturbance to riparian vegetation. Even though the experimental setup and the frequency of floods were different, the riparian disturbances caused by both experiments were similar in nature. Both our experiments suppressed woody plants through frost damage and favored forbs, which led to an increase in species richness (Table 2). Grasses, on the other hand, did not react to the 1-year experiments, probably because they have their budding parts below or close to the ground and remain dormant during winter, which make them less sensitive to winter floods (Engström et al. 2011; Lind and Nilsson unpublished data).

The community composition did not change in any of the experiments even though the species richness increased. Furthermore, there was a decrease in the percent cover of dwarf-shrubs (such as *V. myrtillus*) whereas forbs increased in cover (such as *F. ulmaria*). This suggests that the differences in plant communities along anchor-ice-rich reaches are species specific, with some being favored while others are disfavored. The watering experiments do not include all types of physical disturbance that ice and winter floods might induce on riparian vegetation. For example, ice breakup and ice scouring are not mimicked and could potentially have caused a strong direct effect on plant community composition. Along reaches where plants annually and repeatedly experience flooding or ice events, the effects on community composition are evident, showing the difference between the chronic disturbances versus short-term experiments (Table 3). The reasons for plant responses to winter flooding vary among species. For example, *Vaccinium* spp. were reduced in cover as they are evergreen and sensitive to freezing and may suffer from frost burn if they start photosynthesizing while their basal parts are still being frozen into ice (Bokhorst et al. 2008). A high number of *V. myrtillus* shoots were damaged and reduced in cover by both flooding experiments, opening up patches for forbs to colonize (Fig. 3A). This mechanism could also explain the expansion of *F. ulmaria*. Plants close to the stream channel may also be affected by ice movement and ice jamming during ice breakup and by ice sheets detaching from the riparian zone (Lind et al. 2014). The fact that most wooden sticks were missing along anchor-ice-rich reaches, and in the plots closest to the stream channel, supports this view. Ice mechanically erodes riparian soil and vegetation and thereby favors establishment of new species (Rood et al. 2003). The suggested role of anchor ice is supported by the finding that there was no difference in the number of broken sticks between anchor-ice-rich and anchor-ice-free reaches, as the ice would transport the sticks. Broken sticks were most common in plots farther away from the stream channel and were most likely caused by trampling (by animals or humans), as all broken pieces were still present on the site, and thereby not likely caused by ice movement.

The reason we did not observe any difference in riparian bryophyte cover after the flooding experiments probably reflects the fact that bryophytes are more resistant to freezing than are vascular plants (Minami et al. 2005), suggesting that 1-year experiments are probably too short to have an impact. The observation that anchor-ice-free reaches had a higher cover of riparian bryophytes supports this conclusion. In contrast, in-stream moss transplants showed a clear response to anchor ice, reflecting a more direct effect by ice. This difference may be caused by the canopy-forming stature of *Fontinalis*, making it more sensitive to disturbance. The fact that the *Fontinalis* transplants were dried, and probably less elastic, could also have played a role. According to Muotka and Virtanen (1995), *Fontinalis* species are rather indifferent in their choice of habitat although they form their densest stands at the most stable sites, in this case the anchor-ice-free sites.

We conclude that a dynamic flooding regime during winter, often associated with anchor-ice formation, may be a key component for favoring riparian plant diversity along boreal streams and rivers. The climate change scenarios for this boreal area indicate that ice-induced winter floods will be less likely by the end of the present century, suggesting that the species-rich riparian vegetation in streams and rivers in cold regions will change slowly from forb to dwarf-shrub dominance and that the species richness will decrease accordingly. A common view among researchers is that predicted climate changes will lead many species to shift their distribution limits northward and upward and result in generally higher species richness in currently cold regions (Pauli et al. 2012; Garamvölgyi and Hufnagel 2013). If the special position of riparian zones as being the most species-rich habitats in such regions becomes less pronounced, the future distribution of species richness among habitats in currently cold regions is likely to exhibit novel patterns.
